# LCMV‐specific CD4 T cell dependent polyclonal B‐cell activation upon persistent viral infection is short lived and extrafollicular

**DOI:** 10.1002/eji.201948286

**Published:** 2019-11-27

**Authors:** Ute Greczmiel, Nike J. Kräutler, Mariana Borsa, Alessandro Pedrioli, Ilka Bartsch, Kirsten Richter, Paola Agnellini, Gregor Bedenikovic, Annette Oxenius

**Affiliations:** ^1^ Institute of Microbiology ETH Zürich Zürich Switzerland

**Keywords:** LCMV, CD4 T cells, B cells, polyclonal B cell activation, chronic infection

## Abstract

Persistent virus infections with non‐ or poorly cytopathic viruses are commonly associated with B cell dysregulations. These include the induction of hypergammaglobulinemia and the emergence of virus‐unspecific antibodies. These seemingly unspecific antibody responses interfere with the virus‐specific humoral immunity and contribute to delayed virus control. Whether these virus‐unspecific antibodies are induced in the B cell follicle or at extrafollicular sites and whether one specific CD4 T cell subset is involved in the polyclonal B cell activation is unclear. Here we studied virus‐unrelated IgG antibody responses against self or foreign antigens in the context of persistent lymphocytic choriomeningitis virus (LCMV) infection. We found that the LCMV‐unspecific antibody response is short‐lived and induced predominantly at extrafollicular sites and depends on the presence of LCMV‐specific CD4 T cells. Our data support a scenario in which activated, virus‐specific CD4 T cells provide help to non‐specific B cells at extrafollicular sites, supporting the production of virus unspecific IgG antibodies during persistent viral infection.

## Graphical abstract

Chronic LCMV infection induces an LCMV‐unspecific antibody (IgG) response, which is short‐lived, and is induced predominantly at extrafollicular sites. This unspecific IgG response depends on the presence of LCMV‐specific CD4 T cells.

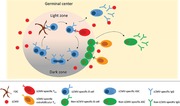

## Introduction

During persistent viral infections with non‐cytopathic viruses like HIV‐1, HCV or HBV in humans or with LCMV in mice, adaptive immunity is significantly altered compared to acute/resolved infections due to continued exposure to high viral antigen burden. CD4 T cell differentiation is markedly skewed towards T follicular helper (T_FH_) cells during such persistent infections 1, 2, 3, 4 and sustained T_FH_ activity is required for eventual control of infection by promoting the late generation of LCMV‐neutralizing antibody responses 5.

One phenomenon antagonizing the appearance of neutralizing antibodies during persistent viral infections is hypergammaglobulinemia; the induction of unusually high levels of IgG titers in serum 6, 7, 8, 9, 10, 11, 12, 13. Hypergammaglobulinemia is a result of the emergence of non‐virus‐specific antibodies, including autoantibodies 7, 9, 14, 15. In persistent LCMV infection, the emergence of unspecific antibodies depends on CD4 T cells and CD40L mediated interaction with unspecific B cells 8, 16. Reducing the overall number of CD4 T cells during persistent infection reduces hypergammaglobulinemia and promotes the appearance of LCMV‐neutralizing antibodies 7, 8. However, the exact kinetics of the LCMV‐unspecific antibody response and whether this response takes place at extrafollicular or follicular sites is unknown. It also remains to be determined whether long‐lived LCMV‐unspecific plasma cells can develop during persistent infection and whether the infection‐induced increase in T_FH_ cells may support these LCMV‐unspecific antibody responses.

To address these questions, we analyzed in detail the LCMV‐unspecific antibody response during persistent LCMV Clone13 (Cl13) infection, focusing on antibody responses against DNP‐OVA or hen egg lysozyme (HEL), as model non‐LCMV related antigens. We discovered that the LCMV‐unspecific antibody response is rather short‐lived and does not involve T_FH_ cells, while depending on CD4 T cells and cognate T:B interactions. Ablation of the immunodominant gp_61‐80_‐specific LCMV‐specific CD4 T‐cell response completely inhibited the appearance of LCMV‐unspecific IgG antibodies. Taken together, the pronounced virus‐specific CD4 T‐cell response during persistent LCMV infection seems to foster the emergence of short‐lived LCMV‐unspecific extrafollicular plasmablasts.

## Results and discussion

### LCMV‐unspecific antibodies are induced during persistent LCMV infection

**Figure 1:**
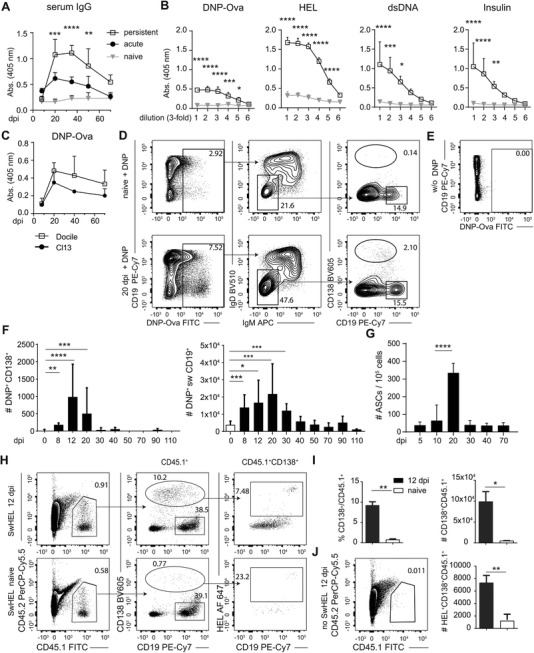
Bystander antibody response in acute and chronic lymphocytic choriomeningitis virus (LCMV) infection. (A) wt B6 mice were acutely or chronically infected with 200 ffu LCMV WE (black filled circles) or 2 × 10^6^ ffu LCMV Cl13 (empty squares). Naïve mice served as controls (grey triangles). Serum was analyzed for total IgG antibodies at indicated time points. (B) DNP‐OVA‐, HEL‐, double stranded (ds) DNA‐ and insulin‐specific IgG antibodies in sera from persistently LCMV Cl13 infected mice on 20 dpi (empty squares) or naïve mice (grey triangles). One representative experiment of two is shown with five mice per group. Statistical analysis done with 2‐way ANOVA, Sidak's multiple comparison test. Data are shown as mean + SD. (C) DNP‐Ova‐specific IgG in sera from mice persistently infected with 2 × 10^6^ ffu LCMV Docile or Cl13 at indicated time points. (D–E) Gating strategy for flow cytometryic analysis of DNP‐OVA‐specific B cells in the spleen 20 dpi after persistent infection LCMV Cl13 compared to naïve mice. +DNP: DNP‐Ova included in staining. (D) DNP^+^ isotype‐switched (IgM^−^IgD^−^) ASC are identified as CD138^+^ CD19^−/low^, total isotype‐switched B cells as CD19^+^. Arrows in the upper panel indicate the gating strategy. Plots were pre‐gated on dump negative (CD4^−^, CD8^−^, Gr‐1^−^, F4/80^−^, NK1.1^−^) lymphocytes. (E) DNP‐OVA gates were set using a staining control without DNP‐OVA. One representative experiment of three is shown with three to five mice per group. (F) Quantification of DNP^+^ ASC and CD19 B cells at different time points post infection. Statistical analysis done with unpaired Mann–Whitney *t*‐test. Data are shown as mean + SD. (G) Quantification of DNP‐Ova specific IgG ASC by ELISPOT in the bone marrow of persistently LCMV Cl13 infected wt B6 mice at indicated days pi. One representative experiment of three is shown with 5 mice per group. Statistical analysis, Mann‐Whitney *U*‐test. Data are shown as mean + SD. (H) Bystander SwHEL (HEL‐specific) B cells were adoptively transferred into persistently infected mice at 4 dpi. Donor‐derived cells were identified as CD45.1^+^, gating into CD138^+^CD19^−/low^ ASC or CD19^+^ B cell and determination of HEL binding in LCMV infected or naïve mice. Arrows in the upper panel indicate the gating strategy. One representative of three mice per group is shown. (I) Quantification of total donor‐derived CD138^+^CD19^−/low^ ASC in frequency and number, as well as the number of HEL^+^ ASCs. Statistical analysis, Welch's *t*‐test. Data are shown as mean + SD. (J) Persistently infected mice with no transferred SwHEL cells served as a negative control for the CD45.1 gating. G‐J: one of two representative experiments is shown. Statistical analysis, Welch's *t*‐test. Data are shown as mean + SD. (A–J) Statistical significance was determined with *(*p* < 0.05), **(*p* < 0.01), ***(*p* < 0.001), and ****(*p* < 0.0001).

We determined the kinetics and the extent of hypergammaglobulinemia during persistent LCMV Cl13 infection by evaluating total IgG levels in serum of infected wt C57BL/6 (B6) mice at different days post‐infection (dpi). Acutely infected mice exhibited moderate and transient increase of total IgG, whereas a more pronounced and sustained IgG increase was seen in chronically infected mice (Fig. 1A). Next, we determined titers of antibodies with specificities for LCMV‐unrelated antigens dinitrophenol‐conjugated OVA peptide (DNP‐OVA), HEL, dsDNA, and insulin in sera from persistently LCMV Cl13 infected wt B6 mice at 20 dpi. Antibodies against all four selected antigens were detected in sera of persistently infected mice (Fig. 1B). Increased levels of DNP‐OVA‐specific IgG antibodies were also observed during chronic infection with LCMV Docile (Fig. 1C). To directly investigate the kinetics and phenotype of DNP‐OVA‐specific B cells during persistent LCMV infection, we identified DNP‐OVA‐specific B cells in spleen (Fig. 1D and E) and bone marrow (BM, Supporting Information Fig. 1A and B) by flow cytometry. The overall number of DNP‐OVA‐specific isotype‐switched CD19^+^ B cells in the spleen expanded until 20 dpi, and thereafter declined to baseline levels by 50 dpi (Fig. 1F). Slightly increased numbers of isotype‐switched DNP‐OVA‐specific B cells were also detectable in BM on 30 dpi, but at much lower numbers compared to spleen (Supporting Information Fig. 1C). In spleen, isotype‐switched DNP‐OVA specific B cells were predominantly CD19^+^CD138^−^ (Fig. 1F), but a small proportion of cells also adopted a plasma cell phenotype (CD138^+^CD19^int/−^, Fig. 1F) and a GC phenotype (CD19^+^CD38^−^Fas^+^, Supporting Information Fig. 1D and E). Quantification of DNP‐OVA specific antibody secreting cells (ASCs) in BM revealed a transient increase, peaking at 20 dpi and returning to baseline levels by 30 dpi (Fig. 1G), further documenting the short‐lived nature of the LCMV‐unspecific IgG response.

We confirmed the activation and differentiation of non‐LCMV‐specific B cells during chronic LCMV infection by adoptively transferring congenically marked (CD45.1) HEL‐specific B cells (SwHEL) into chronically LCMV infected mice. 12 dpi post transfer, a significant proportion of the transferred B cells exhibited a plasma cell phenotype (CD138^+^CD19^int/−^), including those with HEL specificity (Fig. 1H, I and J). However, the proportion of HEL‐binding B cells was markedly reduced within the PC compartment in comparison to the CD19^+^ B cell compartment, indicative of counter‐selection into the PC response in competition with an ongoing LCMV‐specific B‐cell response (Supporting Information Fig. 1F and G).

### LCMV‐unspecific antibodies require LCMV‐specific CD4 T cell help during persistent LCMV infection

The induction of polyclonal B‐cell responses during persistent LCMV infection is dependent on CD4 T cells and cognate interactions between CD4 T cells and B cells 7. We wanted to corroborate that the induction of DNP‐OVA specific antibodies also depends on the presence of CD4 T cells and cognate T and B cell interactions.

**Figure 2:**
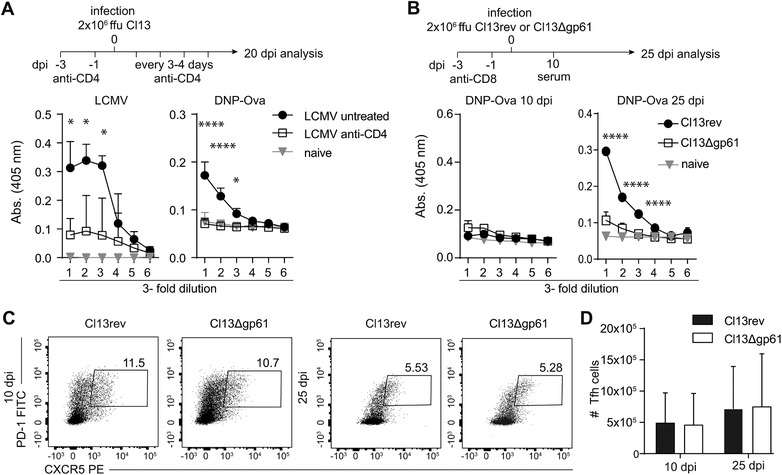
Bystander antibody response in absence of CD4 T cell help. (A) Wt B6 mice were treated with CD4‐depleting antibodies prior and after chronic infection with 2 × 10^6^ ffu LCMV Cl13 at indicated time points and sera analyzed at 20 dpi (empty squares), and compared to a control group without antibody treatment (black, filled circles). Anti‐LCMV and DNP‐OVA IgG was analyzed by ELISA. Naïve sera (grey triangles) served as negative control. One representative of two experiments is shown, three mice per group. (B) Wt B6 mice were transiently depleted of CD8 T cells to allow persistent infection with Cl13Δgp61 (empty squares) or its revertant Cl13rev (black, filled circles). Ten and 25 dpi DNP‐OVA‐specific IgG was determined in serum, naïve sera served as negative control (grey triangles). One representative experiment of three is shown with three mice per experimental group. (C, D) Flow cytometric analysis of T_FH_ frequencies and quantification of total numbers in spleens of Cl13Δgp61 and Cl13rev infected mice at 10 and 25 dpi. Plots pre‐gated on CD4^+^ lymphocytes, one representative experiment of three is shown with three to five mice per experimental group. Statistical analysis in (A–D) was performed with 2‐way ANOVA, Sidak's multiple comparison test. Data are shown as mean + SD. Statistical significance was determined with *(*p* < 0.05), ****(*p* < 0.0001).

First, we infected wt B6 mice with a high dose of LCMV Cl13 and treated half of the group with a CD4 T cell depleting antibody (Fig. 2A), which effectively depleted CD4 T cells in blood and spleen (Supporting Information Fig. 2A and B). As expected, CD4 T‐cell depletion almost completely abrogated induction of DNP‐OVA specific IgG (Fig. 2A), and also HEL‐specific IgG (Supporting Information Fig. 2C) at 20 dpi.

Reducing the magnitude of the LCMV‐specific CD4 T‐cell response was shown to diminish hypergammaglobulinemia, but to increase LCMV specific antibody responses, including neutralizing antibodies, thereby promoting control of the persistent infection 8. We speculated that a reduced LCMV‐specific CD4 T‐cell response might also affect the emergence of LCMV‐unspecific antibodies.

To test this, we employed an LCMV Cl13 mutant with a deletion of the immunodominant CD4 T cell epitope gp_61‐81_ (Cl13Δgp61). Because the LCMV strain Cl13Δgp61 is less virulent, we transiently depleted CD8 T cells in Cl13Δgp61‐infected wt B6 mice to induce a protracted infection and compared the immunological consequences to its revertant (Cl13rev) (Fig. 2B). The Cl13Δgp61 mutant failed to induce a gp_61‐81_ specific CD4 T‐cell response, assessed by adoptive transfer of gp_61‐81_ specific TCR transgenic CD4 T cells (Smarta) (Supporting Information Fig. 2D and E). Thus, Cl13Δgp61 infected mice display an overall LCMV‐specific CD4 T‐cell response that lacks specificity for the gp_61‐81_ epitope.

Strikingly, the induction of DNP‐OVA specific antibodies was completely abolished in Cl13Δgp61 infected mice as opposed to Cl13rev infected mice at 25 dpi (Fig. 2B), also when stratifying for viremic mice only (data not shown). Overall frequencies and numbers of PD1^+^CXCR5^+^ T_FH_ cells were comparable at 10 and 25 dpi in Cl13Δgp61 and Cl13rev infected mice (Fig. 2C and D). Hence, the sole absence of the immunodominant gp_61‐81_ specific CD4 T‐cell response abrogated the induction of LCMV‐unspecific antibodies, while it did not reduce the LCMV‐specific IgG response. If at all, in absence of the gp_61‐81_ specific CD4 T‐cell response LCMV‐specific IgG titers were slightly increased (Supporting Information Fig. 2F and G), resulting in accelerated control of LCMV infection by 35 dpi (Supporting Information Fig. 2H).

Next, we tested whether DNP‐OVA‐specific B cells isolated from chronically LCMV infected mice would present gp_61‐80_ peptide on their MHC class II molecules, enabling them to receive respective T cell help. We implemented a co‐culture with CFSE labelled naïve Smarta CD4 T cells (TCR transgenic CD4 T cells with specificity for gp_61‐80_ peptide) and DNP‐OVA‐specific B cells, either purified from naïve or persistently LCMV Cl13 infected mice on 20 dpi. The percentage of CFSE^low^ Smarta CD4 T cells was significantly higher in co‐cultures with Smarta CD4 T cells and DNP‐OVA‐specific B cells isolated from persistently infected mice compared to naive mice (Supporting Information Fig. 2I and J), suggesting that DNP‐OVA‐specific B cells might present gp_61‐80_ peptide on their surface, rendering them capable of interactions with cognate CD4 T cells. Although the activation of Smarta CD4 T cells by DNP‐specific B cells isolated from chronically LCMV infected mice was rather modest, this observation is in agreement with a previous report documenting presentation of an LCMV NP‐derived peptide on non‐LCMV‐specific B cells 7.

### Emergence of LCMV‐unspecific antibodies is not dependent on the presence of T follicular helper cells

As differentiation of CD4 T cells is markedly skewed towards T_FH_ cells during persistent viral infections like LCMV 1, 2, we reasoned that T_FH_ cells might play a role in the induction of LCMV‐unspecific IgG responses.

**Figure 3:**
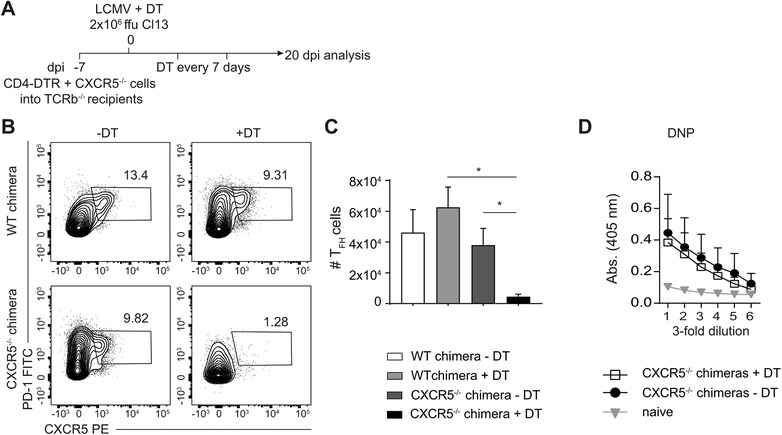
Follicular T help is not required for DNP‐OVA IgG response. (A–C) TCRβ^−/−^ mice were reconstituted with mixed splenocytes from CD4‐DTR and CXCR5^−/−^ donors (CXCR5^−/−^ chimeras), or CD4‐DTR and CD45.1 donors (control chimeras). After 7 days, mice were chronically infected with LCMV Cl13 and treated with DT to ablate CD4‐DTR^+^ T cells at 0, 7, 14 dpi. (B‐C) Ablation of T_FH_ cells was determined in the spleen by flow cytometry (pregated on CD4^+^ lymphocytes) at 20 dpi. One representative of each group is shown, with 3–4 mice per group. Statistical analysis using Welch's *t*‐test. (D) DNP‐OVA‐specific IgG in sera from CD4‐DTR/CXCR5^−/−^ mice treated with or without DT and compared to naïve mice. Statistical analysis, Welch's *t*‐test. Data are shown as mean + SD. One representative of three experiments is shown. Statistical significance was determined with * (*p* < 0.05).

To study whether T_FH_ cells are required for the induction of DNP‐OVA‐specific IgG responses, we used a splenocyte chimera model. TCRβ^−/−^ mice were reconstituted with 50:50 splenocyte mixtures derived from CD4‐diphtheria toxin receptor (DTR) mice and either CD45.1^+^ B6 mice (control chimera) or CXCR5^−/−^ mice (CXCR5^−/−^ chimera) (Fig. 3A). CD4‐DTR mice express the primate DTR in CD4 T cells, allowing their depletion with diphtheria toxin (DT) 5. CXCR5^−/−^ mice cannot form T_FH_ cells as CXCR5 mediates their localization to the B cell follicle 17, 18.

In half of the control and CXCR5^−/−^ chimeras, depletion of the CD4‐DTR derived CD4 T cells was induced before persistent infection with LCMV Cl13, while the rest of the groups remained untreated (Fig. 3A). To control for successful depletion of T_FH_ cells in DT treated CXCR5^−/−^ chimeras, total cell numbers of T_FH_ cells were determined at the end of the experiment in spleen (Fig. 3B and C). On 20 dpi, untreated and DT treated CXCR5^−/−^ chimeras (Fig. 3D) as well as control chimeras (Supporting Information Fig. 3) had mounted DNP‐OVA specific IgG antibodies. The absence of T_FH_ cells from the onset of infection therefore did not impair the emergence of LCMV‐unspecific antibodies. Thus, T_FH_ cells do not play a role in the induction of LCMV‐unspecific antibody responses during chronic infection.

## Concluding remarks

Our data demonstrate that the LCMV‐unspecific antibody response induced in the context of chronic infection is short‐lived and does not rely on T_FH_ cells, but is supported by LCMV‐specific CD4 T cells, as depletion of the immunodominant LCMV‐specific CD4 T‐cell response abrogates the virus‐unspecific IgG response. Reduced unspecific B cell activation results in enhanced virus control, implicating that the virus, among other strategies, might employ the induction of an extensive CD4 T‐cell response to weaken the virus‐specific antibody response and evade recognition.

## Materials and methods

### Mice

C57BL/6 mice were obtained from Janvier Elevage. CD4‐DTR 5, CXCR5^−/−^, CD45.1^+^ B6, TCRβ^−/−^ and CD45.1^+^ Smarta, and SwHEL mice were bred and maintained under SPF conditions at the ETH Phenomics Center.

To create mixed splenocyte chimeras, TCRβ^−/−^ mice received splenocyte mixtures consisting of cells isolated from one quater of donor spleen each. Mice were kept for one week prior to use in experiments to allow engraftment of the cells.

All animal experiments were performed according to institutional guidelines and Swiss federal regulations, and were approved by the veterinary office of the canton of Zürich (animal experimentation permission 147/2014 and 115/2017).

### Adoptive transfer of SWHEL B cells

Recipient mice were infected with 2 × 10^6^ ffu LCMV Cl13 and 4 days later 1 × 10^6^ B cells from CD45.1^+^ SwHEL donors were adoptively transferred. 8 days later, CD45.1^+^ donor‐derived B cells were analyzed by flow cytometry for CD138^+^CD19^−/low^ ASC, CD19^+^ B cells, and HEL binding. Persistently infected mice with no transferred SwHEL cells served as a negative control for the CD45.1 gating.

### Virus and viral peptides

Peptide gp61‐81 (GLNGPDIYKGVYQFKSVEFD) was purchased from NeoMPS. A total of 1 µg/mL of peptide was used to load DCs for 1 h at 37°C.

Strains of LCMV used in this study were LCMV WE, LCMV Docile, LCMV Cl13 wildtype, LCMV Cl13 mutant lacking the gp61 epitope (Cl13Δgp61) and its revertant (Cl13rev). All LCMV Cl13 strains were propagated on BHK‐21 cells and viral titers were determined as described before (Battegay et al., 1991). Mice were infected with 2 × 10^6^ ffu i.v. for persistent infection. Mice were infected with 200 ffu LCMV‐WE or 2 × 10^6^ ffu LCMV Cl13 or LCMV Docile for acute infection and chronic infection, respectively.

### Depletion of CD4 and CD8 T cells

CD4 T cells were depleted by i.p. injection of 600 µg α‐CD4 monoclonal antibody (YTS 191.1; BioXCell) per mouse every 3 days.

CD8 T cells were transiently depleted before persistent infection with C13Δgp61 or C13rev by two i.p. injections of 200 µg α‐CD8 monoclonal antibody (YTS 169.4; BioXCell) per mouse at 3 and 1 day prior to infection.

### ELISA

ELISA was performed using 96‐well Nunc Maxisorp Immunoplates (VWR International AG). Plates were coated overnight at 4°C with the respective specific antigen diluted in 0.1M sodium carbonate buffer (pH = 9.6). For detection of LCMV‐specific antibodies plates were coated with 20 µg/mL lysate of LCMV‐Clone13 infected MC57G cells or of uninfected MC57G cells (negative control). For detection of DNP‐Ovalbumin‐specific antibodies the plates were coated with 30 µg of DNP‐Ovalbumin. For detection of HEL or dsDNA specific antibodies plates were coated with 10 µg/mL solutions of the respective antigen (HEL and dsDNA obtained from Sigma‐Aldrich). Lastly, for detection of insulin‐specific antibodies plates were coated with 50 µg/mL insulin (Sigma‐Aldrich). After coating, plates were blocked with 1% BSA in PBS for 2 h at RT. On a parallel plate, sera were prediluted either 1:40 (LCMV‐specific antibodies), 1:8 (DNP‐Ovalbumin‐ or HEL‐specific antibodies) or 1:5 (dsDNA‐ or Insulin‐specific antibodies) in 1x PBS containing 0.1% BSA and then a threefold dilution series was performed. Then, 100 µL per well of the diluted sera were transferred to the antigen‐coated ELISA plates and incubated for 1 h at RT. Thereafter, plates were incubated for 1 h at RT with an HRPO‐coupled goat anti‐mouse IgG antibody (Sigma) diluted 1:500 in PBS containing 0.1% BSA. Between each step, plates were washed three times with PBS‐T. HRPO was detected by an ABTS color reaction, which was measured 1 h later with a spectrophotometer (Spectramax Plus, Molecular Devices) at a wavelength of 405 nm.

For determination of total serum IgG titers, plates were directly coated with 50 µL of serum obtained from infected or naïve B6 mice. After the blocking step, detection of mouse IgG antibodies was performed.

### ELISPOT

ELISPOT assays for detection of DNP‐Ovalbumin‐specific antibody secreting cells (ASC) in BM were carried out in 96‐well Multiscreen‐IP PVDF Filter Plates (Millipore). Plates were coated for 90 min at 37°C with 30 µg/mL DNP‐OVA diluted in 50 µL 1 × PBS. As negative control, some wells were treated with 1 × PBS alone. After coating, plates were blocked with R10 medium. A total of 1 × 10^5^ BM cells were transferred in 100 µL R10 medium onto the coated ELISPOT plates. As positive control, some wells received instead of cells a 1:1000 dilution of a goat anti‐DNP polyclonal antibody mixture (Jackson Immunoresearch Laboratories). Afterwards, plates were incubated overnight at 37°C without disturbing or moving them. Then, plates were incubated with 50 µL of a biotin‐coupled anti‐mouse IgG antibody (Jackson Immunoresearch Laboratories Inc.) diluted 1:1000 in 1x PBS. Thereafter, the biotinylated antibody was detected with AP‐coupled Streptavidin (Vector Laboratories) diluted 1:1000 in 50 µL of 1x PBS. Eventually detection was performed using the AP Conjugate Substrate Kit (Biorad). Between every step plates were washed six times with PBS‐T. Counting of spots was performed using the AID ELISpot reader (CTL, Germany) with the AID ELISpot software (Version 7.0).

### Flow cytometry and lymphocyte stimulation

Flow cytometry stainings were performed on blood samples, splenocytes and BM cells according to guidelines for the use of flow cytometry in immunological studies 19. Antibodies used for flow cytometry were purchased from BD biosciences (αCD95‐PE and αLy6G‐APC‐Cy7), ebioscience (αCD38‐ PERCP‐Cy5.1, αCXCR5‐PE, and αPD‐1‐FITC), and Biolegend (αCD4‐PB, αCD4‐BV605, αCD8‐PERCP, αCD3‐PE‐Cy7, αCD44‐BV510, αIFN‐γ‐APC, αTNF‐FITC, αCD19‐PE‐Cy7, αCD138‐BV605, αIgD‐BV510, αIgM‐APC, αTer119‐APC‐Cy7, αCD3‐APC‐Cy7, αNK1.1‐APC‐Cy7, αCD11b‐APC‐Cy7, αCD11c‐APC‐Cy7, and αF4/80‐APC‐Cy7). Surface stainings were generally performed at 4°C for 20 min. All samples were treated with 1 mL ACK lysis buffer for 10 min at RT, and fixed in PBS containing 1% PFA.

DNP‐OVA‐specific B cells were stained using DNP‐Ovalbumin coupled to the fluorophore FITC. B cells were surface stained with α‐CD19, α‐CD138, α‐CD95, α‐CD38, α‐IgD, and α‐IgM (BioLegend) at 4°C for 20 min after Fc block. DNP‐Ovalbumin‐specific B cells were stained intracellularly with DNP‐Ovalbumin‐FITC.

Transferred SwHEL cells were characterized by staining with HEL (Sigma), and identified as CD45.1^+^ CD45.2^−^ cells HyHEL9^+^, after blocking with anti‐Fc (CD16/32) (provided by Prof. Robert Brink, 20). B cell subpopulations were further classified by CD138 and CD19 surface staining.

Multiparameter flow analysis was performed on a FACS LSR II flow cytometer (BD) with FACSDiva software. Data analysis was performed with FlowJo software (FlowJo Enterprise, Version 10).

### Statistical analysis

For statistical analysis non‐parametric Mann–Whitney U tests were performed using GraphPad Prism Software. For statistical analysis of ELISA data, in general 2‐way ANOVA, Sidak's multiple comparison test was applied using GraphPad Prism Software. Statistical significance was determined with *(*p* < 0.05), **(*p* < 0.01), ***(*p* < 0.001), and ****(*p* < 0.0001).

## Author contributions

UG and AO designed the experiments; UG, NK, MB, AP, IB, KR, PA and GB performed the experiments; UG, NK, MB, AP, IB, KR and AO analyzed the experiments; UG, NK, AP and MB carried out data and statistical analyses and UG and AO wrote the manuscript.

## Conflict of interest

The authors declare no commercial or financial conflict of interest.

**Abbreviations**
DTR: diphtheria toxin receptor
HEL: hen egg lysozyme

## Supporting information

Supporting Information
