# Supplementary material for: LCMV‐specific CD4 T cell dependent polyclonal B‐cell activation upon persistent viral infection is short lived and extrafollicular
Source: Eur J Immunol. 2019 Nov 27;50(3):396–403. doi: 10.1002/eji.201948286 (PMC7079077; doi:10.1002/eji.201948286)
Supplement: Supplementary file 1 — Supporting Information [file EJI-50-396-s001.pdf]

European Journal of Immunology

Supporting Information

for

DOI 10.1002/eji.201948286

Ute Greczmiel, Nike J. Kräutler, Mariana Borsa, Alessandro Pedrioli, Ilka Bartsch, Kirsten Richter, Paola Agnellini, Gregor Bedenikovic and Annette Oxenius

LCMV-specific CD4 T cell dependent polyclonal B-cell activation upon persistent viral infection is short lived and extrafollicular

© 2019 The Authors. *European Journal of Immunology* published by WILEY-VCH Verlag GmbH & Co. KGaA, Weinheim

Supporting information

Supplemental Figures and Figure legends

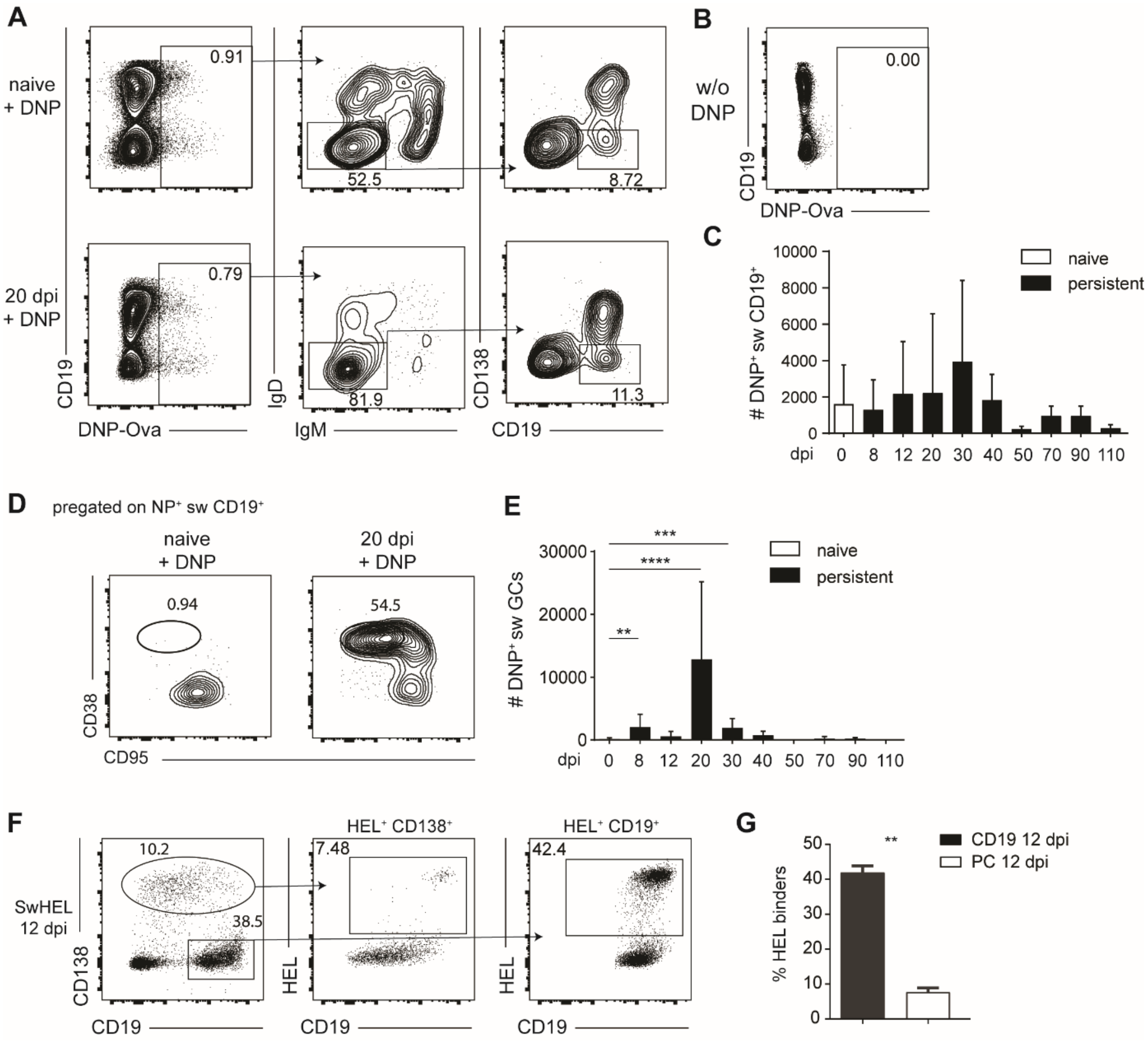

Sup Fig. 1 Characterization of the bystander antibody response in acute and chronic LCMV infections. (A) Flow cytometric analysis of DNP-Ova-specific B cell subsets in the bone marrow 20 dpi post persistent infection with $2x10^6$ ffu LCMV Cl13 compared to naïve mice. Arrows in the upper panel indicate the gating strategy. Plots were pre-gated on dump negative (CD4$^-$, CD8$^-$, Gr-1$^-$, F4/80$^-$, NK1.1$^-$) lymphocytes. Representative plots of 3 experiments with 3-5 mice per group **(B)** DNP-Ova gates were set using a staining control without DNP-Ova. **(C)** Isotype switched DNP$^+$ isotype-switched (IgM$^-$IgD$^-$) B cells were quantified and statistical analysis done by Mann-Whitney t-test. **(D)** Flow cytometric analysis of DNP-Ova-specific B cells in the spleen 20 dpi post persistent infection with $2x10^6$ ffu LCMV Cl13 compared to naïve mice. Further subgating of DNP$^+$CD19$^+$ B cells identified in **Fig. 1** into Fas$^+$CD38$^-$ isotype switched GC. Pooled data from 3 experiments with 3-5 mice per group. **(E)** Quantification of DNP$^+$GC cells in the spleen at indicated time points post infection and compared to naïve levels. **(F-G)** Flow cytometric analysis for the determination of HEL-binders frequency in CD138$^+$CD19$^{-/low}$ ASCs or CD19$^+$ B cells, one representative of 3 mice per group is shown. Arrows indicate the gating strategy. **(G)** Statistical analysis, Welch's t-test.

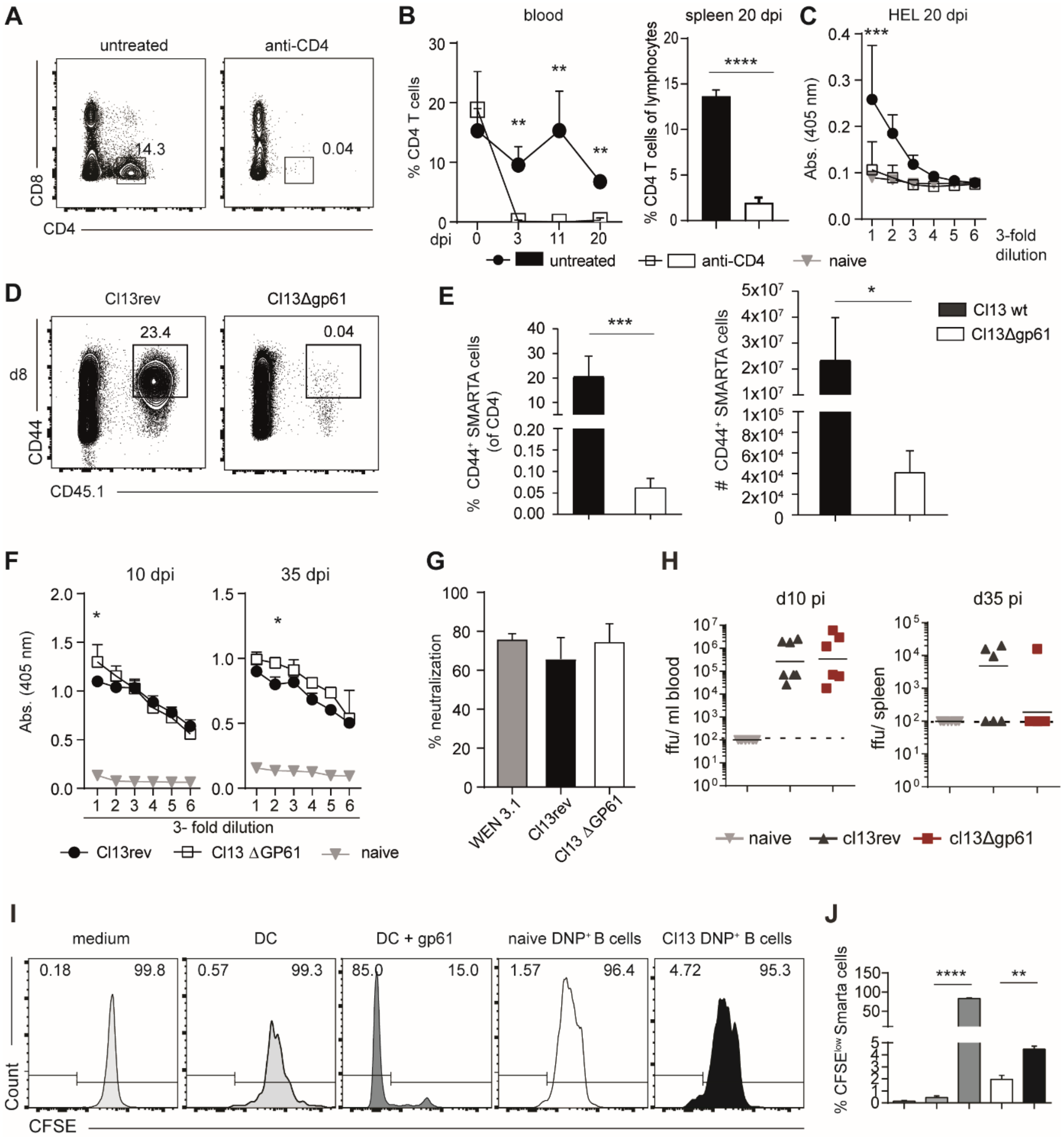

Sup Fig. 2 Characterization of the bystander antibody response in absence of CD4 T cell help. Wt mice were treated with CD4-depleting antibodies prior and after chronic infection with $2x10^6$ ffu LCMV Cl13 at indicated time points as shown in Fig. 2 (A-B). CD4 depletion was assessed by flow cytometry (representative gating shown in A; pre-gated on lymphocytes) at indicated time points post infection (empty squares) and compared to a control group without antibody treatment (black, filled circles). **(B)** Frequency of $CD4^+$ lymphocytes enumerated in blood and spleen at indicated time points. One representative of two experiments is shown, three mice per group. Statistical analysis 2-way Annova, Sidak's multiple comparison test. **(C)** Sera were analyzed for anti-HEL titers at 20 dpi, pre-dilution of sera 1:8, 3-fold dilution series. Naïve sera (light grey arrowheads) served as negative control. One representative of two experiments is shown, three mice per group. Statistical analysis 2-way Anova, Sidak's multiple comparison test. **(D-E)** Wt mice were transferred with $5x10^6$ $CD45.1^+$ SMARTA T cells one day prior to infection with an acute dose (200ffu) of the LCMV Clone13 mutant lacking the immunodominant CD4 T cell epitope gp61 (Cl13Δgp61, empty squares) or its revertant LCMV Clone 13 (Cl13rev), black, filled circles). At 8 dpi the frequency and total numbers of activated SMARTA T cells was determined by flow cytometry. One representative plot is shown, five mice per group. Statistical analysis Welch's t-test. **(F-H)** Wt mice were transiently depleted CD8 T cells to allow persistent infection with the LCMV Clone13 mutant lacking the immunodominant CD4 T cell epitope gp61 (Cl13Δgp61, empty squares) and the response compared to its revertant LCMV Clone 13 (Cl13rev, black, filled circles). **(F)** 10

and 35 dpi antibody titers against LCMV were determined in the sera of these mice, naïve sera served as negative control (light grey arrowheads). Statistical analysis 2-way Annova, Sidak's multiple comparison test. **(G)** Sera taken at 35 dpi and prediluted 1:6 were tested for their ability to neutralize 100 ffu virus and compared to the LCMV neutralizing antibody WEN 3.1 (1.3 μg). Statistical analysis 2Welch's t-test. **(H)** Viral titers were determined in the blood at 10 dpi and in the spleen at 35 dpi. One representative experiment of four is shown with 6-8 mice per experimental group. Statistical analysis 2Welch's t-test. **(I-J)** CFSE-labelled Smarta CD4 T cells were co-cultured for five days either with DCs without (left, light grey) or with gp61 peptide (left center, dark grey) or with DNP-Ova-specific B cells isolated from naïve (center right, white) or from persistently LCMV Clone13 infected mice (20 dpi, right, black). CFSE dilution was analyzed by flow cytometry and the percentage of $CFSE^{low}$ Smarta CD4 T cells was quantified. One representative experiment of two is shown with 3 mice per group. Statistical analysis unpaired Mann-Whitney t-test.

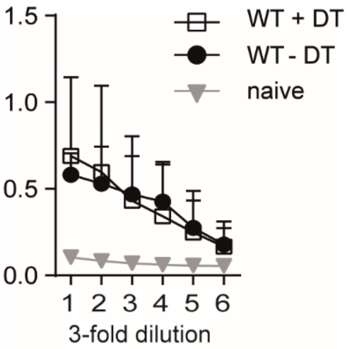

Sup Fig. 3 DT administration does not affect DNP-Ova specific IgG in control chimeras. $TCR\beta^{-/-}$ mice were reconstituted with mixed splenocytes from CD4-DTR and CD45.1 (CD4-DTR/WT) splenocytes. After 7 days mice were infected with $2x10^6$ ffu LCMV Cl13 and treated with DT to ablate CD4-DTR lymphocytes at 0, 7, 14 dpi to ablate $CD4-DTR^+$ T cells. Antibody titers against DNP-Ova were tested at 20 dpi in sera from CD4-DTR/WT chimeras, treated or not treated with DT and compared to naïve mice; sera were prediluted 1:8, 3-fold dilution series. Statistical analysis, Welch's t-test. One representative of three experiments is shown.

Supplemental Materials and Methods

Preparation of DNP-Ovalbumin

For coupling of DNP to Ovalbumin, 27.6 mg of Ovalbumin were dissolved in 5 ml of a 0.15 M K_2CO_3 solution while gently stirring the solution. Then 100 mg of 2,4-dinitrobenzene sodium sulfonate (DNBS) were dissolved in 1ml of pre-warmed (37°C) ddH_2O. The DNBS solution was then added slowly, drop-by-drop to the Ovalbumin solution. Afterwards, the reaction was covered with foil to prevent exposure to light and gently stirred at RT o/n. The next day, dialysis was performed to exchange the K_2CO_3 solution with a 0.001 M phosphate buffer in a 3ml Slide-A-Lyzer™ Dialysis Cassette (10K MWCO; Thermo-Scientific) for two to three days with two to three buffer exchanges per day. Afterwards, the DNP-Ovalbumin solution was removed from the dialysis cassette and centrifuged at full speed for 20 min at 4°C to remove precipitates. Then, the concentration of DNP-Ovalbumin was determined in the supernatant using a NanoDrop. DNP-Ovalbumin was afterwards stored in small aliquots at -20°C. Once an aliquot was thawed, re-freezing of the remaining solution was avoided. Instead the aliquot was kept at 4°C and exposure to light was avoided. At 4°C DNP-Ovalbumin was stable for about two weeks.

Coupling of DNP-Ovalbumin to Fluorescein isothiocyanate (FITC) for flow cytometry stainings

1mg of DNP-Ovalbumin was first dialysed, and thereby also concentrated, against a FITC coupling buffer (0.05 M boric acid, 0.2M NaCl, pH 9.2) using a Pierce™ Protein Concentrator PES (10K MWCO; Thermo- Scientific) tube. Dialysis and concentration was performed by centrifugation for 20 minutes at 4°C at 4000xg as described in the manufactures protocol. After each centrifugation step the flow-through was discarded and new coupling buffer was added to the dialysis chamber. After the last centrifugation step, DNP-Ovalbumin (now contained in ca. 100 µl of the FITC-coupling buffer) was removed from the dialysis chamber and incubated with 10µl of a freshly prepared 1mg/ml Fluorescein (Sigma-Aldrich) in dimethylsulphoxid (DMSO) solution for 1h at 37°C in the dark (waterbath). Afterwards, DNP-Ovalbumin-FITC was purified using a Disposable PD 10 Desalting Column (GE Healthcare) following the manufacturer's protocol. DNP-Ovalbumin-FITC was concentrated using a Pierce™ Protein Concentrator PES (10K MWCO; Thermo- Scientific) tube by centrifugation for 20 min at 4°C and 4000xg. The concentrated DNP-Ovalbumin-FITC was stored at 4°C in the dark and used for flow cytometry analysis. As DNP-Ovalbumin is only stable for two weeks at 4°C, DNP-Ovalbumin-FITC needed to be prepared regularly.

CFSE labelling and co-culture of Smarta CD4 T cells and DNP-Ovalbumin-specific B cells

DNP-Ovalbumin- specific B cells were obtained by FACS sorting from naïve or persistently LCMV-Clone13 infected mice on d20 pi. Single cell suspensions of splenocytes were produced and treated for

3 min with ACK lysis buffer to dissolve erythrocytes. Surface staining of DNP-Ovalbumin-specific B cells was performed with DNP-Ovalbumin-FITC for 30 min at RT in the dark in 1xPBS containing 10%FCS and 2mM EDTA (Sorting buffer). After a washing step with sorting buffer cells were purity FACS sorted using a FACS Aria (BD) with FACSDiva software.

At the same time, Smarta CD4 T cells were negatively selected by MACS (Miltenyi Biotech). At first single cell suspensions from Smarta spleens were obtained as before. Then suspensions were incubated with a mixture of biotinylated antibodies against CD19, CD11b, Ly-6C and Dx5 (all purchased from BD bioscience) diluted 1:100 in sorting buffer for 20 min at 4°C. After a washing step, the cell pellets were then incubated with a mixture of different MACS microbeads (Miltenyi Biotech) against CD8, CD11c and Biotin diluted 1:100 in sorting buffer for 20 min at 4°C. After another washing step, negative selection was performed using a MS MACS column (Miltenyi Biotech) following the manufacturer's instructions.

After MACS sorting, obtained CD4 T cells were labelled with CFSE (Sigma-Aldrich). At first, cells were resuspended in 2ml RPMI medium containing 10% FCS (R10 medium) and then were incubated for 2 min at 37°C in a water bath. Then, a 5mM CFSE solution was added in a 1:1 ratio to the cells and the mixture was incubated for further 12 min at 37°C. Afterwards, the cells were washed twice with ice-cold R10 medium and then incubated for 10 min in 14ml of R10 medium to quench the reaction. Afterwards, cells were centrifuged for 5min at 1600 rpm at 4°C once more and resuspended in R10 medium. The cell number was determined using a Neubauer counting chamber.

For co-culture, 10^4 DNP-Ovalbumin- specific B cells and $6x10^4$ CFSE labelled Smarta CD4 T cells were seeded into a 96-U-bottom well plate (1:6 ratio). As a negative control, Smarta CD4 T cells were seeded in wells of the 96-well plate alone. As positive control, $6x10^4$ CFSE-labelled Smarta CD4 T cells were seeded with 10^4 DCs which were either unloaded or loaded with gp61 peptide. Afterwards, cells were co-cultured for 5 days in a cell incubator. Thereafter, CFSE dilution in Smarta CD4 T cells was measured by flow cytometry on a FACS LSR II flow cytometer (BD) with FACSDiva software. Data analysis was performed with FlowJo software (FlowJo Enterprise, Version 10).

LCMV-neutralization assay

Neutralizing capacities of sera were tested in a foci-reduction assay. Sera were first heat-treated at 56°C for 10 min to kill all viral particles that might be contained in the sera. Afterwards, sera were diluted 1:4 in Modified Eagles Medium (MEM) containing 2% FCS in a 96-well plate and a 2-fold dilution series was performed. Then 50 foci-forming units of the respective virus inoculum was added to the serum dilutions and incubated for 90 min at 37°C in a cell incubator. Afterwards 200 µl of the serum-virus mixtures were transferred onto 200 µl of a MC57G solution (concentration: 8 x 10^5 cells/ml) seeded in 24-well plates. Afterwards the plates were incubated for two to four hrs in a cell incubator

to allow infection of cells. Thereafter circa 200µl of a viscous medium, consisting of 1:1 mixed 2% methylcellulose and 2-times Dulbecco Modified Eagles Medium (DMEM) containing 10% FCS, was added on top of every well to prevent spread of newly formed viral particles by infected cells. Plates were incubated for 48 hours at 37°C before fixation of the cell layer and visualization of the foci as described elsewhere (Battegay *et al.*, 1991). Neutralization capacities of sera were determined by reference to the number of foci that were counted when MC57G cells were infected with virus which was not pre-incubated with serum or infected with virus pre-incubated with naïve serum.
